# The use of research evidence on patient preferences in pharmaceutical coverage decisions and clinical practice guideline development: exploratory study into current state of play and potential barriers

**DOI:** 10.1186/s12913-014-0540-2

**Published:** 2014-11-11

**Authors:** Cecile MA Utens, Trudy van der Weijden, Manuela A Joore, Carmen D Dirksen

**Affiliations:** Clinical Epidemiology and Medical Technology Assessment, Maastricht University Medical Centre, P.O. Box 5800, 6202 AZ Maastricht, The Netherlands; CAPHRI, School for Public Health and Primary Care, Faculty of Health, Medicine and Life Sciences, Maastricht University, P.O. Box 616, 6200 MD Maastricht, the Netherlands; CAPHRI, School for Public Health and Primary Care, Department of Family Medicine, Faculty of Health, Medicine and Life Sciences, Maastricht University, P.O. Box 616, 6200 MD Maastricht, the Netherlands

**Keywords:** Patient preference, Pharmaceutical coverage, Clinical practice guideline

## Abstract

**Background:**

The patient perspective is increasingly considered in healthcare policy decisions. The use of research on patient preferences seems however limited. Using the available research on patient preferences would make healthcare policy decisions more evidence-based regarding the patient perspective. Objective of this study is to investigate whether and how results of research on patient preferences are incorporated in current procedures for pharmaceutical coverage decisions and clinical practice guideline (CPG) development.

**Methods:**

A document analysis on procedure descriptions was combined with case studies. Analyses were performed for five European countries. In the document analysis we systematically checked whether the procedure provides guidance on the systematic use of research on patient preferences, and whether the search and use of research on patient preferences is mentioned in the decision making procedure. In the case studies, which were for exploratory purposes, we scored whether or not research question on patient preferences were formulated, whether or not a search strategy including terms relating to patient preferences was mentioned, whether results of this search strategy were shown and finally, how many references with preference-related terms were included in the reference list of the case.

**Results:**

None of the procedures for pharmaceutical coverage decisions mentions the systematic consideration of research on patient preferences. For CPG development, the Scottish procedure refers to a mandatory literature search. In the Netherlands this step is optional. In the case studies for pharmaceutical coverage decisions only one reference related to patient preferences was found. Some of the case studies for CPG included research questions, search strategies and references relating to patient preferences.

**Conclusions:**

This study illustrates that systematic consideration of research on patient preferences in pharmaceutical coverage decisions and guideline development is limited, or if taken into account, this is not visible. This contrasts the strong movement towards patient involvement in health care. Several potential barriers may explain the limited use of research on patient preferences.

**Electronic supplementary material:**

The online version of this article (doi:10.1186/s12913-014-0540-2) contains supplementary material, which is available to authorized users.

## Background

There is an increasing body of empirical research on patient preferences towards health care and healthcare outcomes [1]. However, the use of this research in healthcare policy decision making seems limited [2-5]. Much of the literature on the importance of incorporating the patient perspective in healthcare policy decisions focuses on direct, active participation (e.g. membership of committees) [6]. However, it is questioned whether active participation is the best way of incorporating the patient perspective into healthcare policy decisions [7]. Using research on patient preferences is another way for incorporating the patient perspective in healthcare policy decisions, which may contribute to the evidence-base of active patient participation [7,8]. This article contributes to the literature on incorporating the patient perspective in healthcare policy decisions by focusing on the use of research on patient preferences. In this paper we focus on two types of decisions: pharmaceutical coverage decisions (i.e. uptake of removal of a pharmaceutical in the benefit package) and clinical practice guideline (CPG) development. Integration of research evidence on patient preferences in pharmaceutical coverage decisions and CPG is important for several reasons. A first argument is that research evidence on patient preferences adds to other knowledge bases and is therefore important for decision making. Both pharmaceutical coverage decisions and CPG are – at least in part – evidence-based and informed by research on safety, effectiveness and/or cost-effectiveness. Likewise, using the available research on patient preferences would make healthcare policy-making more evidence-based with regard to the patient perspective. In addition, studies on patient preferences may report on the relative value of outcomes and experiences, which is not covered by most patient reported outcomes and experience measures used in clinical studies. Further, incorporation of research on patient preferences may foster more patient-centeredness of health care in general [6], and even so on the individual level (shared decision making). Finally, when constructing quality adjusted life years (QALYs), the general public’s valuation is used for patients’ health states. As patient preferences may differ from preferences of the general public, the patient perspective gets short shrift. Second argument is that research evidence on patient preferences may serve as a source of information for patient representatives in healthcare decision making (empowerment) and provide what representatives bring to the table with more scientific foundation. A final argument is that patient preferences, amongst others, are known to influence the cost-effectiveness of healthcare services [9]. Furthermore, currently CPG do not always reflect patients’ preferences [10] which causes low adherence to and low acceptability of guidelines [11]. Thus, consideration of research evidence patient preferences in healthcare decision making may improve the quality of decisions and yield return on investment on research on patient preferences.

This paper describes the first step of the Patient-VIP study (Patient Values In Policy making) [12], which is an explorative study on the integration of research on patient preferences in pharmaceutical coverage decisions and CPG. In three sub studies the Patient-VIP study aims to develop a decision framework for the integration of evidence on patient preferences in pharmaceutical coverage decisions and CPG. The objective of the current paper is to explore whether and how such research is incorporated in coverage decision procedures and CPG development. We applied a two-step analysis for this study. First we studied the written guidance for pharmaceutical coverage decisions and CPG development for their content on using research on patient preferences (theoretical perspective). Second we studied specific cases of pharmaceutical coverage decisions and CPGs in order to explore if results of research on patient preferences were considered (practical perspective). We performed both steps for five European countries, namely the Netherlands, England & Wales, Scotland, Germany and France. We choose the Netherlands because it is the origin of the Patient-VIP study. England & Wales, Scotland (as representatives of the United Kingdom), Germany and France were chosen because they are neighbour countries of the Netherlands and the three largest economies in Europe. We also choose these countries because of relevant differences with respect to the funding system of the healthcare system (tax-funded in England & Wales and Scotland versus social insurance-based in France, the Netherlands and Germany). In addition, the chosen countries have leading organisations in guideline development, which will ascertain the availability of literature. Studying the integration of evidence on patient preferences in different countries will reveal similarities and/or differences between countries, which provide valuable information when working towards systematic integration of evidence on patient preferences in pharmaceutical coverage decisions and CPG.

## Methods

### Terminology

Preferences usually refer to the desirability of something or someone [5]. There are several definitions of the term ‘preference’ used in different fields [5]. However, the terminology used for preferences is not unambiguous [3,13]. In the healthcare literature several related terms are used when referring to preferences for healthcare services or outcomes. These terms include patient perspective [13,14], value, utility [5,7], well-being, perception [13], expectations [3-5,7,14], satisfaction [3,7], experience [4,7,13], goals [3,14], concerns [5], desires [5], needs, view [13], acceptability [13], beliefs [4,13], attitudes [4,13] and quality of life [7]. For this paper we considered all terms mentioned above as (relating to) preferences.

### Data collection

#### Document analysis

In the first step written guidance documents for pharmaceutical coverage decisions and CPG development were studied for their content on consideration of research on patient preferences. Aim was to demonstrate whether and how existing research on patient preferences is incorporated in current procedures for pharmaceutical coverage decisions and CPG development. Documents describing these procedures can be considered as grey literature, as they most likely will not be found through searches in literature databases, like PubMed. Therefore, literature and documents describing current procedures of pharmaceutical coverage decisions and CPG development were identified through internet searches on the websites of the responsible national and international organisations or institutes. Additional file 1: Table S1 lists the organisations of which the websites have been searched. In addition, reference lists of retrieved documents were hand searched for additional information on the procedures. The references that were searched are listed in Additional file 2. In the guidance documents we systematically checked: 1) whether the procedure provides guidance on the systematic consideration of research on patient preferences; and 2) whether the search for and use of research on patient preferences is mentioned in the decision making procedure.

#### Case studies

We studied two pharmaceutical coverage decisions and two CPG for each country in order to explore whether research on patient preferences was considered. The case studies are labour intensive. Because this study is exploratory, we limited the number of case studies to two per type of decision-making. With two case studies it is still possible to see whether the results are similar or deviating. In general, the cases were selected after a discussion of the authors which revealed that for these cases multiple treatment options are available, and patients’ could have preferences for either option.

The pharmaceutical coverage decisions chosen were biologicals for treating rheumatoid arthritis (available for all five countries) [15-20] and pemetrexed for the first-line treatment of non-small-cell lung cancer (not available for Germany) [21-24]. This was also a pragmatic choice as it turned out to be difficult to find matching pharmaceutical coverage decisions for all countries.

For the case studies for CPG we were able to identify matching and representative guidelines for all counties on only one topic: rheumatoid arthritis [25-29]. The second case study for each country differed between the countries and were the treatment of prostate carcinoma for the Netherlands [30], treatment of generalised anxiety disorder for England & Wales [31], treatment of congestive heart failure for Scotland and France [32,33] and treatment of depression for Germany [34]. These guidelines were selected because a PubMed search showed that research on patient preferences was published at the moment of development. The general and country-specific search was performed by combining the different terms relating to ‘preference’ with the guideline topic. For each guideline and coverage decision we searched if and how evidence on patient preferences was used by: 1) checking whether or not research/scoping questions on patient preferences were formulated; 2) checking if a search strategy was mentioned including terms related to patient preferences; 3) investigating whether the results of the search strategy were displayed; 4) scoring how many reference titles of the reference list included terms that related to patient preferences; and for pharmaceutical coverage decisions 5) whether the decision text holds information relating to research on patient preferences”. The terms searched for and scored were derived from previous studies, see above. Additional terms were discussed between the authors.

#### Member check

A member check on the results of the document analysis with representatives of the organisations involved in the procedures was performed in order to assure that we included all relevant documents and whether we interpreted the documents correctly. We sent representatives the paper by mail and asked for their comments. In total we approached 14 persons and received six reactions. These reactions concerned the coverage decision procedures in the Netherlands, England & Wales, Scotland and Germany and the CPG development procedures of England & Wales, Scotland and Germany. The member check revealed additional literature on recent developments in Germany’s pharmaceutical coverage decisions. The member check for Dutch pharmaceutical coverage decisions required only small changes in the procedure description and not in the interpretation of the documents. The member check on German CPG pointed out additional information which was incorporated in this contribution. The other member checks did not result in additional literature and our interpretation was correct. Overall, the member check did not alter our conclusions.

## Results

### Pharmaceutical coverage decisions –documents analysis on procedure and patient preferences

Additional file 3: Table S2 provides an overview of the organisations involved in pharmaceutical coverage decisions and their procedures. None of the countries’ procedures mentions the systematic search for and use of research on patient preferences, but the guidance documents leave room for optionally using preference-related aspects. In the Netherlands the procedure mentions that “in a wider consideration of effectiveness, aspects like … quality of life are discussed” (p. 37) [35]. The Dutch procedure also mentions that “softer end-points like quality of life, patient satisfaction and experiences of patients .... are considered in the review” (p. 11) [36]. In England & Wales, quality of life is an outcome measure used for the appraisal (p. 14). In England & Wales, patient and carer groups can make evidence submissions which may provide perspectives from patients on experiences, preferences and expectations (p. 23) [37]. The procedure states that “standard qualitative research techniques, such as thematic analysis, facilitate the synthesis of evidence of this type. Accounts and experiences may be collected and analysed systematically using these qualitative research techniques, but there is no requirement to present the information in this way” (p. 23) [37]. In Scotland, patient interest groups can make information submissions, which may include “patient views on treatments and medicines” (p. 1) and “preferences” (p. 1) [38]. The scientific requirements for this information are not further stated. The procedure in Germany mentions that “legal requirements specify inclusion of the following benefit parameters in the assessment: .... improvement in the quality of life” (p. 518) [39]. This is to be defined in the scoping prior to the assessment. The economic evaluations of England & Wales, Scotland and the Netherlands include QALYs as an outcome, which are based on population preferences for quality of life outcomes [38,40,41]. As population values may differ from patient values this was not considered as fulfilling our inclusion criteria for research on patient preferences. The French and German procedures do not mention QALYs, neither based on patient, nor on population preferences for health outcomes [41].

### Clinical practice guidelines- documents analysis on procedure and patient preferences

The different procedures of CPG development can be found in Additional file 4: Table S3.

The development procedure in Scotland mentions “a literature search strategy to identify both qualitative and quantitative studies that reflect patients’ experiences and preferences in relation to the clinical topic” (p. 19) that is mandatory and should take place before the first meeting of the working committee [42]. In the Netherlands this is an optional step: “literature search into the patient perspective … is recommended to be performed prior to the guideline development” (p. 7) [43]. Furthermore, the Dutch procedures also mention “specific literature search to the needs and/or preferences of patients” (chapter 3.2) [44]. If findings from these studies are incorporated in CPG, they are often reported as ‘other considerations’ right below the recommendations, or in a separate generic chapter of the guideline. In the development procedure of England & Wales “patient experience …should inform development of a structured review question. In addition review questions that focus on a specific element of patient experience may merit consideration in their own right.” (p. 59) [45]. Consequently, if one of the review questions is focused on patient preferences, a literature search for evidence concerning the topic will be performed. On what is exactly meant by patient experiences, the guideline states: “Patient experience....covers a wide range of dimensions, including: patient views on the effectiveness and acceptability of given interventions; patient preferences for different treatment options and patient views on what constitutes a desired, appropriate or acceptable outcome”. (p. 65) [45]. The key role of patient and carer members of guideline development groups in England & Wales is “raising awareness of grey literature known to them (for example patient surveys) that highlights patient issues…” (p. 46) [45]. The guideline development group in England & Wales is “encouraged to actively search for qualitative research on patients’ views and experiences…” (p. 20) [46]. The English/Welsh and Scottish procedures also mention that patient groups and local organisations are invited to submit evidence or information on patient views [42,45]. The German procedure does not mention the search for research on patient preferences, but patient organisations are asked to work up and document patient experiences and are provided with tools to do so [personal communication from member check CS]. The French procedures do not make any reference to the consideration of research on patient preferences [41].

### Case studies

The first coverage decision studied was on biologicals for rheumatoid arthritis and/or ankylosing spondylitis. None of the pharmaceutical coverage decisions mentioned research questions or a search strategy regarding patient preferences. The Scottish and English/Welsh coverage decision includes a reference with the term quality of life in the title [15,16,24]. The texts of the Dutch, English/Welsh and Scottish pharmaceutical coverage decisions include passages referring to research findings on patients’ quality of life [15,16,22]. The Dutch, English/Welsh and Scottish documents report on QALYs based on population preferences for health outcomes [15,16,22-24]. In the Scottish document it is stated that patient interest groups made a submission, without providing details [16].

The second coverage decision studied was on pemetrexed for non-small lung cancer. None of the pharmaceutical coverage decisions’ texts mentioned research questions or a search strategy regarding patient preferences, nor did the reference lists include references relating to research on patient preferences. In the English/Welsh, Scottish and Dutch reports a reference is made to a study that elicited population preferences for health-related quality of life in lung cancer by a visual analogue scale and a standard gamble technique interview [17,19,20]. No reference to patient preferences for health-related quality of life is made [17,19,20]. The Dutch and French reports state that evidence on the effects on quality of life are not available [17,19]. The Scottish report mentions that a submission by a patient interest group was received, but no details regarding this submission are provided [20].

Table 1 shows the results for the two CPG case studies. The use of research on patient preferences varied between the guidelines and between countries. A research question on patient preferences does not necessarily lead to higher percentages of references with preference-related terms in the guideline. Table 2 shows how many references were found in the guidelines on rheumatoid arthritis, per term. The terms that are mostly mentioned in the reference lists are “(health related) quality of life”, “patient perspective” and “well-being”. Table 2 shows how many references were found in the guidelines that differed between the countries. The terms that appeared most often were “quality of life” and “needs for”.

**Table 1:** **Results for the clinical practice guideline case studies**

	**Rheumatoid arthritis**				
	**The Netherlands**	**England/Wales**	**Scotland**	**Germany**	**France**
Were research questions set on patient preferences?	No	Yes	No	No	Yes
Was search strategy described?	No	No	Yes, prior to development	No	No
Were results of search strategy summarised?	No	No	Yes, presented to development group	No	No
Number of references with preference-related term in reference list (% of total number of references)	12 (1.9%)	34 (8%)	1 (1%)	8 (3%)	5 (1%)
	**The Netherlands – Prostate carcinoma**	**England/Wales – Generalised anxiety disorder**	**Scotland –Chronic heart failure**	**Germany - Depression**	**France – Chronic heart failure**
Were research questions set on patient preferences?	Yes	Yes	No	Yes	No
Was search strategy described?	No	Yes	No	No	No
Were results of search strategy summarised?	No	No	No	No	No
Number of references with preference-related term in reference list (% of total number of references)	48 (5%)	26 (6%)	8 (5%)	13 (1%)	0

**Table 2:** **Number of references including specific preference-related term in the different guideline cases**

	**England & Wales GAD case**	**Scotland CHF case**	**Netherlands Prostate CA case**	**France CHF case**	**Germany Depression case**	**England & Wales RA case**	**Scotland RA case**	**Netherlands RA case**	**France RA case**	**Germany RA case**	**Total**
Value	0	0	0	0	0	0	0	0	0	0	0
Utilities	1	0	0	0	0	0	0	2	0	1	4
Preference	3	1	5	0	0	2	1	0	0	0	12
View	1	0	1	0	0	1	0	0	0	0	3
Goal	0	0	0	0	0	0	0	0	0	0	0
Belief	3	0	0	0	0	1	0	0	0	0	4
Expectation	0	0	0	0	0	0	0	0	0	0	0
Experience	2	0	5	0	0	2	0	0	0	0	9
Satisfaction	0	0	0	0	0	3	0	1	0	0	4
Health related quality of life	3	1	3	0	1	9	0	2	2	1	22
Quality of life	4	3	18	0	3	4	0	2	0	1	35
Concern	0	1	2	0	0	0	0	0	0	0	3
Desire	0	0	0	0	0	0	0	0	0	0	0
Perspective	2	0	4	0	3	2	0	4	1	0	16
Attitude	0	0	0	0	1	0	0	0	0	0	1
Perception	1	1	0	0	0	1	0	0	0	0	3
Well being	1	1	1	0	1	4	0	1	1	1	11
Need (for)	5	0	9	0	4	2	0	0	0	0	20
Choice	0	0	0	0	0	2	0	0	0	1	3
PRO	0	0	0	0	0	1	0	0	0	3	4

GAD: generalised anxiety disorder; CA: carcinoma; RA: Rheumatoid Arthritis.

## Discussion

The results of this explorative study confirm our expectation that systematic consideration of research on patient preferences is limited in both pharmaceutical coverage decisions and CPG (clinical practice guideline) development, or if it is considered, this is not visible. This result is more strongly present in pharmaceutical coverage decisions than in CPG development. The coverage case studies showed that research on patient preferences were mainly found in the documents text as research findings on quality of life. The CPG guidance documents indicate that the consideration of research on patient preferences is optional (the Netherlands), not mandatory but encouraged (England & Wales) or completely absent (France and Germany). Only the Scottish procedure explicitly mentions a “search for patient evidence”, which is executed before the start of the CPG development and is informative for the development of review questions. Several CPG cases reported research questions relating to patient preferences, for which a literature search was performed, in a way comparable to other research questions. The case studies on CPG showed several references with terms related to preferences. The terms most frequently used were “quality of life”, “needs”, “perspective” and “well-being”.

The absence of instructions for the consideration of results of research on patient preferences in pharmaceutical coverage decisions confirms the limited focus on this type of research in health technology assessment [3]. In CPG development the importance of incorporating research on patient preferences is more acknowledged, as could be derived from the results of the case studies. The GRADE classification system for the strength of recommendations in guidelines, holds that patient preferences, among others, can affect the strength of recommendations [47]. Our results on the consideration of research on patient preferences for CPGs compare well with a recent Dutch study, which found that in 13 out of 62 guidelines an attempt was made to use research on patient preferences [6].

The limited attention for the consideration of research on patient preferences, especially in pharmaceutical coverage decisions, is in contrast with the strong movement towards patient involvement in healthcare decision making. This suggest that there may be barriers for the consideration of research on patient preferences in pharmaceutical coverage decisions and CPG development. Literature mentions several barriers that have normative origin, conceptual origins, methodological origins, procedural origin and practical origin. Normative barrier is that HTA and health economics community, the predominant measure in economic evaluation is the Quality Adjusted Life Year (QALY), which is determined by using preferences of a general population for health states of patients. Brazier et al. performed a review mentioning all positive, normative and methodological arguments both in favour and against the use of patient preferences [48]. Arguments relate for example to the allocation of collective resources, the foundations of welfare economics, as well as systematic differences between public and patient values. Second normative barrier is that the QALY is derived from patient outcomes for quality of life related to health. However, patients may derive utility from aspects beyond health, e.g. process or organisation of care, which is purposely excluded from the QALY [49]. It should be determined whether society is willing to pay for the preferences beyond health outcomes. If we decide to incorporate these preferences, the methodological issues of the limitations of the QALY should be addressed. Procedural barrier is that the place of evidence on patient preferences in pharmaceutical coverage decisions and CPG development is not well determined. There is debate on the potential benefits and downsides of the integration of evidence on patient preferences. In the field of CPG development some consider using research on patient preferences as an unsubstantiated add on to already lengthy and complex guidelines that may further decrease adherence, usability and possibilities for transfer of knowledge into translate into practice [50]. Some view patient preferences as fundamentally a property of the individual and question the usefulness of aggregate preferences for individual decision making [3,50]. Indeed, patient preferences can be very heterogeneous. However, current decision making is also informed by aggregate data on effectiveness and cost-effectiveness. Aggregate data on patient preferences are however not a substitute for elicitation of individual preferences when clinicians discuss treatment choices with their patients. Contrary, aggregate evidence on patient preferences may indicate that decisions are preference-sensitive [3,5] which may stimulate clinicians to help patients construct their individuel preferences. In addition, research in general supports individual decision making, as more information may reduce uncertainty. Other procedural barrier is that the importance attached to evidence on patient preferences is, purposely, not equal to that placed on evidence on effectiveness and cost-effectiveness [3,51]. It remains unclear whether this type of evidence should get some weight in pharmaceutical coverage decisions and guideline development, and how this relates to other evidence.

Barrier of conceptual origin is that previous studies showed that there is much variability in terminology used for preferences [14]. This is confirmed in our study in the procedures for guideline development and demonstrated in the CPG case studies. We retrieved several discussion papers on the subject of integration of patient preferences in healthcare decision making, using a wide spectrum of terms. In addition to the ambiguity in terminology, there is heterogeneity in the type of study design and measurement techniques that constitute preference related research [3,13]. Furthermore, it is stated that evidence on patient preferences is often of low quality and shows variability in results [5]. It is argued that the variability in terminology and different measuring methods result in difficulties in the search for these studies, the synthesis of evidence and the judgement whether the results are valid for the purpose of pharmaceutical coverage decisions and CPG. Some authors argue that there is a lack on research on patient preferences, which is considered a practical barrier [50,51]. Indeed, at the time of pharmaceutical coverage decisions are made research on patient preferences is often not available. However, for CPG it is not clear whether there is indeed an absolute lack on this type of evidence or that the available research is not recognised as such. Finally, a methodological barrier is that it is argued that there are no clear existing methods for incorporating evidence on patient preferences in the decision- or development process [51].

Before systematic integration of research on patient preferences in the procedures of pharmaceutical coverage decisions and CPG development will succeed, the previously described issues need to be addressed. The lack of conceptual and methodological clarity in preference research can be addressed by the development of a conceptual map on preferences, incorporating all contributions from all relevant disciplines and a taxonomy to systematically categorise the different types of research (and measurement techniques), as suggested by Brooker et al. [13] and Chong et al. [3]. The procedural issues need to be addressed by reaching consensus on the place of patient preferences in decision making and addressing possible barriers for integration. To address both issues we currently undertake a qualitative study as was prescribed in a previously published research protocol [12]. This qualitative study constitutes interviews with relevant stakeholders in which the opinions and ideas of relevant stakeholders with regard to the facilitators and barriers for integrating research on patient preferences in pharmaceutical coverage decisions and CPG, as well as the taxonomy for research on patient preferences values, will be addressed. The results of the current study will be used as input for the qualitative study and the subsequent development of a taxonomy for research on patient preferences.

Our study has several limitations. We only studied written documents for answering our research question in this explorative study. Written guidance documents provide guidance on how procedures should be executed and the written pharmaceutical coverage decisions and CPG are the result of the procedure that was followed. We therefore considered them a reliable source of information. We acknowledge however that there may be discrepancies between what is written down and the practical execution that was not reported in the coverage decision or CPG, as decision making can be seen as a dynamic process which is sensitive to contextual factors. Exploring this possible discrepancy requires other research methodologies and was beyond the scope of this paper.

Unfortunately, we did not receive a member check response for both pharmaceutical coverage decisions and CPG for all countries. As a result, we may have missed valuable additional information. However, the responses we did receive did not change our conclusions. We therefore do not expect that additional responses would have changed our conclusions.

Despite the member check, and although we have thoroughly reviewed the national procedures of pharmaceutical coverage decisions and CPG development on the integration of research values, it is possible that we missed documents that may have included more information on this subject. The number of case studies we performed is not sufficient to draw firm conclusions. Our results are at best indicative for current practice., The focus of our study was the use of results of research on patient preferences (passive patient participation) and not on other forms of (active) patient participation (e.g. membership of CPG development group). In the member check the confusion on the difference between active participation and the consideration of research on patient preferences became clear. Indeed we do realise that patient participation is a broad concept which has many forms. Much is already known on active participation in pharmaceutical coverage decisions and CPG development and we intentionally focussed on the consideration of research as little is known on this topic. However, there is a grey area when defining research. Research includes existing, secondary research, but it can be debated whether it also includes results of primary research (e.g. focus groups) performed for the purpose of the specific coverage decision or guideline under development. Another limitation may be that although we included a broad range of terms that referred to patient preferences, this range may have been incomplete, and we may have missed relevant terms. Nonetheless, this would mean the variability in terms used to refer to patient preferences is even larger than presumed, it emphasises the need for more uniformity and transparency in terminology.

## Conclusions

In current written procedures for pharmaceutical coverage decisions there does not seem to be room for the consideration of research on patient preferences. In clinical practice guidelines there is more attention for patient preferences research, although not in a systematic manner. We have listed several barriers that need to be addressed before research on patient preferences can systematically be considered in pharmaceutical coverage decisions and clinical practice guidelines. Future research should elaborate more on the barriers and how to overcome them. We have planned to do so in the upcoming steps of the patient-VIP study.

## Additional files

Additional file 1: Table S1. List of organisations included in the website searching. Additional file 2:
**Documents for coverage decisions.**
Additional file 3: Table S2. Procedure of coverage decisions in 5 European countries. Additional file 4: Table S3. Procedure of clinical practice guideline development in 5 European countries.
