# Supplementary material for: The use of research evidence on patient preferences in pharmaceutical coverage decisions and clinical practice guideline development: exploratory study into current state of play and potential barriers
Source: BMC Health Serv Res. 2014 Nov 11;14:540. doi: 10.1186/s12913-014-0540-2 (PMC4229609; doi:10.1186/s12913-014-0540-2)
Supplement: Additional file 1: Table S1. — List of organisations included in the website searching. [file 12913_2014_540_MOESM1_ESM.doc]

Table S1. List of organisations included in the website searching

| **The Netherlands** |  | **England** |  |
| --- | --- | --- | --- |
| *Coverage* | *Clinical Practice Guidelines* | *Coverage* | *Clinical Practice Guidelines* |
| College voor zorgverzekeraars (CVZ) | Regieraad Kwaliteit van Zorg | National Institute for Health and Clinical Excellence (NICE) | National Institute for Health and Clinical Excellence (NICE) |
|  | Dutch Association of Medical Specialists |  |  |
|  | The Dutch College of General Practitioners (NHG) |  |  |
|  | Dutch Institute for Healthcare Improvement (CBO) |  |  |
| **Germany** |  | **Scotland** |  |
| *Coverage* | *Clinical Practice Guidelines* | *Coverage* | *Clinical Practice Guidelines* |
| Der Gemeinsame Bundesausschuss  **(**G**-**BA) | Agency for Quality in Medicine (ÄZQ) | Scottish Medicines Consortium (SMC) | Scottish Intercollegiate Guidelines  Network (SIGN) |
| Institute for quality and efficiency in health care (IQWIG) | Association of the Scientific Medical Societies in Germany (AWMF) |  |  |
| **France** |  | **General** |  |
| *Coverage* | *Clinical Practice Guidelines* | *Coverage* | *Clinical Practice Guidelines* |
| Haute Autorité de Santé (HAS) | Haute Autorité de Santé (HAS) | ISPOR global health care systems road map | Guidelines International Network |
