# Supplementary material for: The use of research evidence on patient preferences in pharmaceutical coverage decisions and clinical practice guideline development: exploratory study into current state of play and potential barriers
Source: BMC Health Serv Res. 2014 Nov 11;14:540. doi: 10.1186/s12913-014-0540-2 (PMC4229609; doi:10.1186/s12913-014-0540-2)
Supplement: Additional file 2: — Documents for coverage decisions. [file 12913_2014_540_MOESM2_ESM.doc]

Documents for coverage decisions

**The Netherlands:**

Mastenbroek CG, van der Meer FM, Zwaap J, Rikken F, Polman P. Pakketbeheer in de praktijk. Diemen: College voor zorgverzekeringen; 2006.

Staal PCL, G. Beoordeling stand van de wetenschap en praktijk. Diemen: College Voor Zorgverzekeraars; 2007.

van der Meijden CG, C. Procedure beoordeling extramurale geneesmiddelen: Ministerie van Volksgezondheid, Welzijn en Sport en College Voor Zorgverzekeraars; 2011.

Zwaap J. Pakketbeheer in de praktijk 2. Diemen: College voor zorgverzekeraars; 2009.

**England & Wales:**

National Institute for Health and Clinical Excellence (NICE). Contributing to a technology appraisal - a guide for patient/carer groups. London: National Institute for Health and Clinical Excellence; 2004.

National Institute for Health and Clinical Excellence (NICE). Patient and public involvement policy.

National Institute for Health and Clinical Excellence (NICE). Guide to the methods of technology appraisal. London: National Institute for Health and Clinical Excellence; 2013.

All Wales Medicines Strategy Group AWMS. AWMSG summary guidelines for appraising medicines. 2012 [cited 10-9-2013]; Available from: http://www.awmsg.org/awmsgonline/docs/awmsg/appraisaldocs/inforandforms/AWMSG%20summary%20guidelines%20for%20appraising%20medicines.pdf

All Wales Medicines Strategy Group. A flow chart outlining the appraisal principles and process. 2012 [cited 10-9-2013]; Available from: http://www.awmsg.org/awmsgonline/docs/awmsg/appraisaldocs/inforandforms/AWMSG%20appraisal%20principles%20and%20process%20flowchart.pdf

**Scotland:**

Scottish Medicines Consortium. [cited 6-9-2013]; Available from: <http://www.nice.org.uk/mediacentre/factsheets/ScottishMedicinesConsortium.jsp>

Scottish Medical Consortium (SMC). Working with SMC - A guide for Manufacturers. Glasgow: Scottish Medical Consortium.

Scottish Medical Consortium (SMC). Patient Interest Group - Submission Guidance and Template. Glasgow: Scottish Medical Consortium; 2012.

**Germany:**

Bekkering GE, Kleijnen J. Procedures and methods of benefit assessments for medicines in Germany. Eur J Health Econ. 2008; 9 Suppl 1:5-29.

Gemeinsamer Bundesausschuss (G-BA). Assessment of the benefits of pharmaceuticals according to

s. 35a SGB V.

Gemeinsamer Bundesausschuss (G-BA). [cited 22-5-2012];

Available from: http://www.english.g-ba.de/legalduties/procedures/

Gemeinsamer Bundesausschuss (G-BA). Geschäftsordnung des Gemeinsamen Bundesausschuss (code of procedure); 2012.

Gemeinsamer Bundesausschuss (G-BA). Therapiehinweise gemäß § 92 Abs. 2 Satz 7 SGB V i. V. m.

§ 17 AM-RL zur wirtschaftlichen Verordnungsweise von Artzneimitteln; 2012.

**France:**

Haute Autorite de Sante (HAS). General method for assessing health technologies. [cited 4-10-2013]; Available from: http://www.has-sante.fr/portail/upload/docs/application/pdf/general_method_eval_techno.pdf.

Haute Autorite de Sante (HAS). Framework for cooperation with associations of patients and users. [cited 4-10-2013]; Available from: http://www.has-sante.fr/portail/upload/docs/application/pdf/2010-09/2e20guide20coopc3a9ration20assoc.patients20gb.pdf

**Multiple countries:**

Special issue: Health care technology and its assessment in eight countries: Australia, Canada, France, Germany, Netherlands, Sweden, United Kingdom, United States. Health Policy. 1994; 30(1-3):1-421.

Stafinski T, Menon D, Davis C, McCabe C. Role of centralized review processes for making reimbursement decisions on new health technologies in Europe. Clinicoecon Outcomes Res. 2011;3:117-86.

**Documents for Clinical Practice Guidelines**

**The Netherlands:**

Broerse J, van der Ham L, van Veen S, Pittens C, van Tulder M. Inventarisatie patientenparticipatie bij richtlijnontwikkeling. Amsterdam: Athena Instituut, Vrije Universiteit Amsterdam; 2010.

Kwaliteitsinstituut voor de gezondheidszorg CBO. Evidence-based richtlijn ontwikkeling handleiding voor werkgroepleden; 2007.

Kwaliteitsinstituut voor de gezondheidszorg CBO. Evidence-based richtlijn ontwikkeling handleiding voor werkgroepleden; 2007.

Kwaliteitsinstituut voor de gezondheidszorg CBO, Nederlandse Patiënten Consumenten Federatie. Blauwdruk patientenparticipatie in richtlijnonwikkeling. Leidraad voor richtlijnontwikkelaars en werkgroepleden; 2009.

Raad Kwaliteit - Adviescommissie Richtlijnen. Medisch specialistische richtlijnen 2.[cited 4-10-2013]; Available from: [http://www.kwaliteitskoepel.nl/assets/structured-files/2012/Richtlijn%202_Opmaak%205.pdf](http://www.kwaliteitskoepel.nl/assets/structured-files/2012/Richtlijn 2_Opmaak 5.pdf)

Regieraad Kwaliteit van Zorg. Richtlijn voor richtlijnen. Den Haag: Regieraad kwaliteit van zorg; 2011.

van Veenendaal, H.; Franx, G.C.; Grol, M.H. van Vuuren, J. Versluijs, M.; Dekhuijzen, P.N.R.

Patientenparticipatie in richtlijnontwikkeling. In: van Everdigen JJEB, J.S.; Assendelft, W.J.J.; Swinkels, J.A.; van Barneveld, T.A.; van de Klundert, J.L.M. Evidence-based richtlijnontwikkeling, een leidraad voor de praktijk. Den Haag: Bohn Stafleu Van Loghum; 2004. p. 48-62.

**England & Wales**

National Institute for Health and Clinical Excellence (NICE) . The guidelines manual. London: National Institute for Health and Clinical Excellence; 2012.

National Institute for Health and Clinical Excellence (NICE). Social value judgements, principles for the development of NICE guidelines. [cited 4-10-2013]; Available from: http://www.nice.org.uk/media/C18/30/SVJ2PUBLICATION2008.pdf.

National Institute for Health and Clinical Excellence (NICE). Contributing to a NICE clinical guideline: a guide for patients and carers. London: National Institute for Health and Clinical Excellence; 2006.

National Institute for Health and Clinical Excellence (NICE). A guide for patients and carers contributing to a NICE clinical guideline. London: National Institute for Health and Clinical Excellence; 2006.

Thomas V. Patient and carer involvement in NICE clinical guidelines. .

**Scotland:**

Scottish Intercollegiate Guidelines Network (SIGN). Sign 100 a handbook for patient and carer representatives. Edinburgh: Scottish Intercollegiate Guidelines Network; 2008.

Scottish Intercollegiate Guidelines Network (SIGN). SIGN 50 a guideline developers handbook; 2011.

**Germany:**

AWMF, ÄZQ. Das Leitlinien-manual von AWMF und ÄZQ. Z ärtzl Fortbild Qual sich (ZaeFQ). 2001(95):Suppl 1.

Das Ärztliche Zentrum für Qualität in der Medizin (ÄZQ). Handbuch zur Entwicklung regionaler Leitlinien. Berlin: Aertzliches Zentrum fur Qualitat in der Medizin; 2006.

Das Ärztliche Zentrum für Qualität in der Medizin (ÄZQ). Handbuch Patientbeteiligung. Beteiligung am Programm fur Nationale VersorgungsLeitlinien. Berlin: Artzliches Zentrum fur Qualitat in der Medizin; 2008.

**France:**

Haute Autorite de Sante (HAS). Preparing doctors' guides and lists of procedures and services for chronic conditions. [cited 4-10-2013]; Available from: http://www.has-sante.fr/portail/upload/docs/application/pdf/methode_guide_ald_traduit.pdf.

Haute Autorite de Sante (HAS). Rapid assessment for assessing medical and surgical procedures. [cited 4-10-2013]; Availale from: http://www.has-sante.fr/portail/upload/docs/application/pdf/rapid_assessment_method_eval_actes.pdf.

Haute Autorite de Sante (HAS). Élaboration de recommandations de bonne pratique. Note de cadrage; 2010.

Haute Autorite de Sante (HAS). Élaboration de recommandations de bonne praqtique. Méthode «recommendandations pour la praqtique clinique»; 2010.

**Global:**

World Health Organization (WHO). WHO handbook for guideline development; 2008.
