# Supplementary material for: The use of research evidence on patient preferences in pharmaceutical coverage decisions and clinical practice guideline development: exploratory study into current state of play and potential barriers
Source: BMC Health Serv Res. 2014 Nov 11;14:540. doi: 10.1186/s12913-014-0540-2 (PMC4229609; doi:10.1186/s12913-014-0540-2)
Supplement: Additional file 3: Table S2. — Procedure of coverage decisions in 5 European countries. [file 12913_2014_540_MOESM3_ESM.doc]

Table S2. Procedure of coverage decisions in 5 European countries

|  | the Netherlands | England & Wales | Germany | Scotland | France |
| --- | --- | --- | --- | --- | --- |
| Organisations/ institutes involved | Minister of Health (final  decision-maker)  Health Care Insurance  Board (CVZ)  - Assessment Committee on Pharmaceuticals ( CHF)  - Appraisal Committee  (ACP) | England:  Department of Health requests  appraisals from:  National Institute for Health and  Care Excellence (NICE)  - Technology Appraisal  Committee (TAC)  - Independent Evidence Review  Group (STA) / assessment  group (MTA)  Wales*:  New Medicines Group (NMG)  All Wales Medicines Strategy  Group (AWMSG) | Department of health (approval or disapproval  of decisions)  G-BA joint committee with  subcommittees (decisor)  G-BA office with sub  departments  Institute for quality and  efficiency in health care  (IQWIG) | NHS Scotland (decisions)  Scottish Medicines  Consortium (SMC)  New Drugs Committee  (NDC) | Ministry for health and  social security (decisor)  Haute Autorité de Santé  (HAS) (recommandations)  Commission d’Evaluation Medicaments (CEM)  (former Transparency  Committee) |
| Current coverage decisions procedure and important topics | Procedure [1, 2-5]:  1. Submission by  manufacturer  2. Evaluation report made  based on manufacturer  submission, own literature  search and optional  consultation of  stakeholders and experts 🡪 report on therapeutic value, cost-effectiveness and budget-impact.  3.CFH reviews report and  consults manufacturer and  stakeholders 🡪 revised  report  4.Commenting on revised  report by manufacturer  and stakeholders  5. Optional: appraisal by  appraisal committee 🡪 report with evidence on necessity, effectiveness, cost-effectiveness and feasibility  6. Final advice to minister  by CVZ | Procedure of NICE [3, 6]:  1.Topic selection  2. Scoping  3. Evidence submission by  manufacturer  4. External review group  Reviews evidence submitted  by manufacturer (STA) and  performs own literature search  (MTA), reviews information  and evidence received from  stakeholders and patient expert  and clinical expert. 🡪  evaluation report  5.TAC reviews evaluation  report and hears patient and  clinical expert 🡪 draft  recommendation  6. Commenting by stakeholders  7. Final report  Procedure of AWMSG [13, 14]:  1. Submission by manufacturer.  2. AWMSG steering group  confirm full or limited  submission required.  3. Full or limited submission by  manufacturer.  4. Review by NMG. Review  evidence on effectiveness and  cost-effectiveness.  5. AWMSG final appraisal  Recommendation, takes into  account equity, budget-impact  and broader societal impact.  6. Ministerial ratification  7. Positive or negative  guidance  * The majority of appraisals comes from NICE. AWMSG takes into account the future work programme of NICE and will not consider appraising a product of NICE intends to publish their final appraisal within marketing authorisation date. Advice is interim to NICE advice should this be subsequently published. | Procedure [3,7,8]:  1. Topic selection  2. Defining scope  3. Public comments on scope  4. Assessment of evidence (=  search, appraisal and analysis  of evidence), this step can be  commissioned from external  institutes (IQWiG)  5. Publication of preliminary  report  6. Public commenting on  preliminary report  7.Recommendations and final  report  8. G-BA joint committee  adopts directive  9. Legal supervision by federal  ministry of health  Since January 2011 the AMNOG-law most assessments when introducing new pharmaceuticals follow the following procedure:  1. Evidence submission by  manufacturer, showing the  added benefit.  2. IQWIG evaluation as in 4.  3. G-BA verdict on benefit  grading  4. Reference pricing | Procedure [3, 9-11]:  1. Submission by  manufacturer  2. evaluation report made  3. NDC evaluates report  for SMC 🡪  recommendation draft  4. Comments from  manufacturer to SMC  5. Submissions to SMC  possible from patient  groups  6. Review of comments en  submissions from 4 & 5  7. SMC final  recommendation report  MTAs of NICE in England & Wales are reviewed by NHS Quality Improvement Scotland and, if applicable, deemed to apply within the Scottish context. | Procedure STA [3, 12]:  1 .Submission  manufacturer  2. Evaluation of the report  based on manufacturer’s  submission  3. External review by  experts  4. Medical benefit  appraisal by CEM +  decision Minister of  Health  6. Appraisal of  improvement  of medical benefit +  benefit classification  7. Final reimbursement  decision by Minister of  health  Procedure MTA [3,12]  1. Topic selection by HAS  2. Scoping  3. Literature search and  evaluation 🡪 assessment  report  4. Internal and external  review assessment report  5. Commenting assessment  report by stakeholders  6. consultation  stakeholders in work  group meeting  7. appraisal by sub  committee 🡪  recommendations  8. HAS board approved  recommendation |
| STA: Single Technology Assessment, assessment of technology for 1 indication; MTA: Multiple Technology Assessment, assessment of >1 technology or technology for >1 indication; QALY: Quality Adjusted Life Year | | | | | |

References

1. Mastenbroek CG, van der Meer FM, Zwaap J, Rikken F, Polman P. Pakketbeheer in de praktijk. Diemen: College voor zorgverzekeringen; 2006.
2. Staal PCL, G. Beoordeling stand van de wetenschap en praktijk. Diemen: College Voor Zorgverzekeraars; 2007.
3. Stafinski T, Menon D, Davis C, McCabe C. Role of centralized review processes for making reimbursement decisions on new health technologies in Europe. Clinicoecon Outcomes Res. 2011;3:117-86.
4. van der Meijden CG, C. Procedure beoordeling extramurale geneesmiddelen: Ministerie van Volksgezondheid, Welzijn en Sport en College Voor Zorgverzekeraars; 2011.
5. Zwaap J. Pakketbeheer in de praktijk 2. Diemen: College voor zorgverzekeraars; 2009.
6. National Institute for Health and Clinical Excellence (NICE). Guide to the methods of technology appraisal. London: National Institute for Health and Clinical Excellence; 2013.
7. Gemeinsamer Bundesausschuss (G-BA). Geschäftsordnung des Gemeinsamen Bundesausschuss (code of procedure); 2012.
8. Gemeinsamer Bundesausschuss (G-BA). Therapiehinweise gemäß § 92 Abs. 2 Satz 7 SGB V i. V. m. § 17 AM-RL zur wirtschaftlichen Verordnungsweise von Artzneimitteln; 2012.
9. Scottish Medicines Consortium. [cited 6-9-2013]; Available from: <http://www.nice.org.uk/mediacentre/factsheets/ScottishMedicinesConsortium.jsp>
10. Scottish Medical Consortium (SMC). Working with SMC - A guide for Manufacturers. Glasgow: Scottish Medical Consortium.
11. Scottish Medical Consortium (SMC). Patient Interest Group - Submission Guidance and Template. Glasgow: Scottish Medical Consortium; 2012.
12. Haute Autorite de Sante (HAS). General method for assessing health technologies. [cited 4-10-2013]; Available from: <http://www.has-sante.fr/portail/upload/docs/application/pdf/general_method_eval_techno.pdf>.
13. All Wales Medicines Strategy Group AWMS. AWMSG summary guidelines for appraising medicines. 2012 [cited 10-9-2013]; Available from: http://www.awmsg.org/awmsgonline/docs/awmsg/appraisaldocs/inforandforms/AWMSG%20summary%20guidelines%20for%20appraising%20medicines.pdf
14. All Wales Medicines Strategy Group. A flow chart outlining the appraisal principles and process. 2012 [cited 10-9-2013]; Available from: http://www.awmsg.org/awmsgonline/docs/awmsg/appraisaldocs/inforandforms/AWMSG%20appraisal%20principles%20and%20process%20flowchart.pdf
