# Supplementary material for: The use of research evidence on patient preferences in pharmaceutical coverage decisions and clinical practice guideline development: exploratory study into current state of play and potential barriers
Source: BMC Health Serv Res. 2014 Nov 11;14:540. doi: 10.1186/s12913-014-0540-2 (PMC4229609; doi:10.1186/s12913-014-0540-2)
Supplement: Additional file 4: Table S3. — Procedure of clinical practice guideline development in 5 European countries. [file 12913_2014_540_MOESM4_ESM.doc]

Table S3. Procedure of clinical practice guideline development in 5 European countries

|  | the Netherlands | England & Wales* | Germany | Scotland* | France |
| --- | --- | --- | --- | --- | --- |
| Organisations/  institutes involved | Not coordinated on national level. Among others: CBO, Orde Medisch Specialisten, TRIMBOS, NHG, Regieraad Kwaliteit van Zorg | Department of Health  Centre for Clinical Practice (CCP) of NICE  National Collaborating  Centres (NCCs)  - Guideline Development  Groups (GDG) | ÄZQ  AWMF  Not coordinated on national level, medical societies develop own guidelines | SIGN (Scottish  Intercollegiate Guidelines  Network | Haute Autorité de Santé |
| Current CPG development  procedure and important topics | Procedure [1-3]:  1. Topic selection  2. Working group  selection  3. Analysis of problem and  determine research  questions  4. Literature search and  review  5.Writing draft guideline  6. Comment phase on draft  guideline  7. Authorisation | Procedure [4-8]:  1. Topic referral  2. Scope: determine  framework, draft research  questions and identify  issues, scope prepared by  NCC  3. Literature search and  review by NCC and  GDG  4.Writing draft guideline  (GDG)  5. Consultation on draft  guideline  6. Final guideline  7. Guidance issued | Procedure [9]:  1. Topic selection  2. Working group and  author group selection  3. Determine research  questions  4. Literature search  5. Review literature and  seek consensus  6.Writing draft guideline  7. Consultation round of  experts  8. Pilot testing  9. Draw final guideline  10. Guideline publication | Procedure [10, 11]:  1. Topic selection  2. Literature search on patient  evidence  3. GDG selection and  define remit guideline  4. Define research  questions  5. Literature search and  appraisal  6. Draft guideline  7. National open meeting  for presentation and  discussion 🡪 rewrite  8. Peer review 🡪 rewrite  9. Review editorial group  10. Publication and  dissemination | Procedure [12-16]  1. Literature search and  analysis  2. Writing draft  guideline  3. Consultation of  professional experts  4. Writing draft  guideline  5. Consultation of peer  review group  6. Writing final  guideline  7. Validation guideline  and opinion of HAS  board  8. Publication and  distribution |

References

1. Broerse J, van der Ham L, van Veen S, Pittens C, van Tulder M. Inventarisatie patientenparticipatie bij richtlijnontwikkeling. Amsterdam: Athena Instituut, Vrije Universiteit Amsterdam; 2010.
2. Raad Kwaliteit - Adviescommissie Richtlijnen. Medisch specialistische richtlijnen 2.[cited 4-10-2013]; Available from: [http://www.kwaliteitskoepel.nl/assets/structured-files/2012/Richtlijn%202_Opmaak%205.pdf](http://www.kwaliteitskoepel.nl/assets/structured-files/2012/Richtlijn 2_Opmaak 5.pdf)
3. Regieraad Kwaliteit van Zorg. Richtlijn voor richtlijnen. Den Haag: Regieraad kwaliteit van zorg; 2011.
4. National Institute for Health and Clinical Excellence (NICE). Patient and public involvement policy.
5. National Institute for Health and Clinical Excellence (NICE) . The guidelines manual. London: National Institute for Health and Clinical Excellence; 2012.
6. National Institute for Health and Clinical Excellence (NICE). Social value judgements, principles for the development of NICE guidelines. [cited 4-10-2013]; Available from: http://www.nice.org.uk/media/C18/30/SVJ2PUBLICATION2008.pdf.
7. National Institute for Health and Clinical Excellence (NICE). Contributing to a NICE clinical guideline: a guide for patients and carers. London: National Institute for Health and Clinical Excellence; 2006.
8. National Institute for Health and Clinical Excellence (NICE). A guide for patients and carers contributing to a NICE clinical guideline. London: National Institute for Health and Clinical Excellence; 2006.
9. AWMF;, ÄZQ. Das Leitlinien-manual von AWMF und ÄZQ. Z ärtzl Fortbild Qual sich (ZaeFQ). 2001(95):Suppl 1.
10. Scottish Intercollegiate Guidelines Network (SIGN). Sign 100 a handbook for patient and carer representatives. Edinburgh: Scottish Intercollegiate Guidelines Network; 2008.
11. Scottish Intercollegiate Guidelines Network (SIGN). SIGN 50 a guideline developers handbook; 2011.
12. Haute Autorite de Sante (HAS). Framework for cooperation with associations of patients and users. [cited 4-10-2013]; Available from: http://www.has-sante.fr/portail/upload/docs/application/pdf/2010-09/2e20guide20coopc3a9ration20assoc.patients20gb.pdf
13. Haute Autorite de Sante (HAS). Preparing doctors' guides and lists of procedures and services for chronic conditions. [cited 4-10-2013]; Available from: http://www.has-sante.fr/portail/upload/docs/application/pdf/methode_guide_ald_traduit.pdf.
14. Haute Autorite de Sante (HAS). Rapid assessment for assessing medical and surgical procedures. [cited 4-10-2013]; Availale from: http://www.has-sante.fr/portail/upload/docs/application/pdf/rapid_assessment_method_eval_actes.pdf.
15. Haute Autorite de Sante (HAS). Élaboration de recommandations de bonne pratique. Note de cadrage; 2010.
16. Haute Autorite de Sante (HAS). Élaboration de recommandations de bonne praqtique. Méthode «recommendandations pour la praqtique clinique»; 2010.
